# Supplementary figures and images for: Interleukin-10 control of pre-miR155 maturation involves CELF2
Source: PLoS One. 2020 Apr 23;15(4):e0231639. doi: 10.1371/journal.pone.0231639 (PMC7179890; doi:10.1371/journal.pone.0231639)

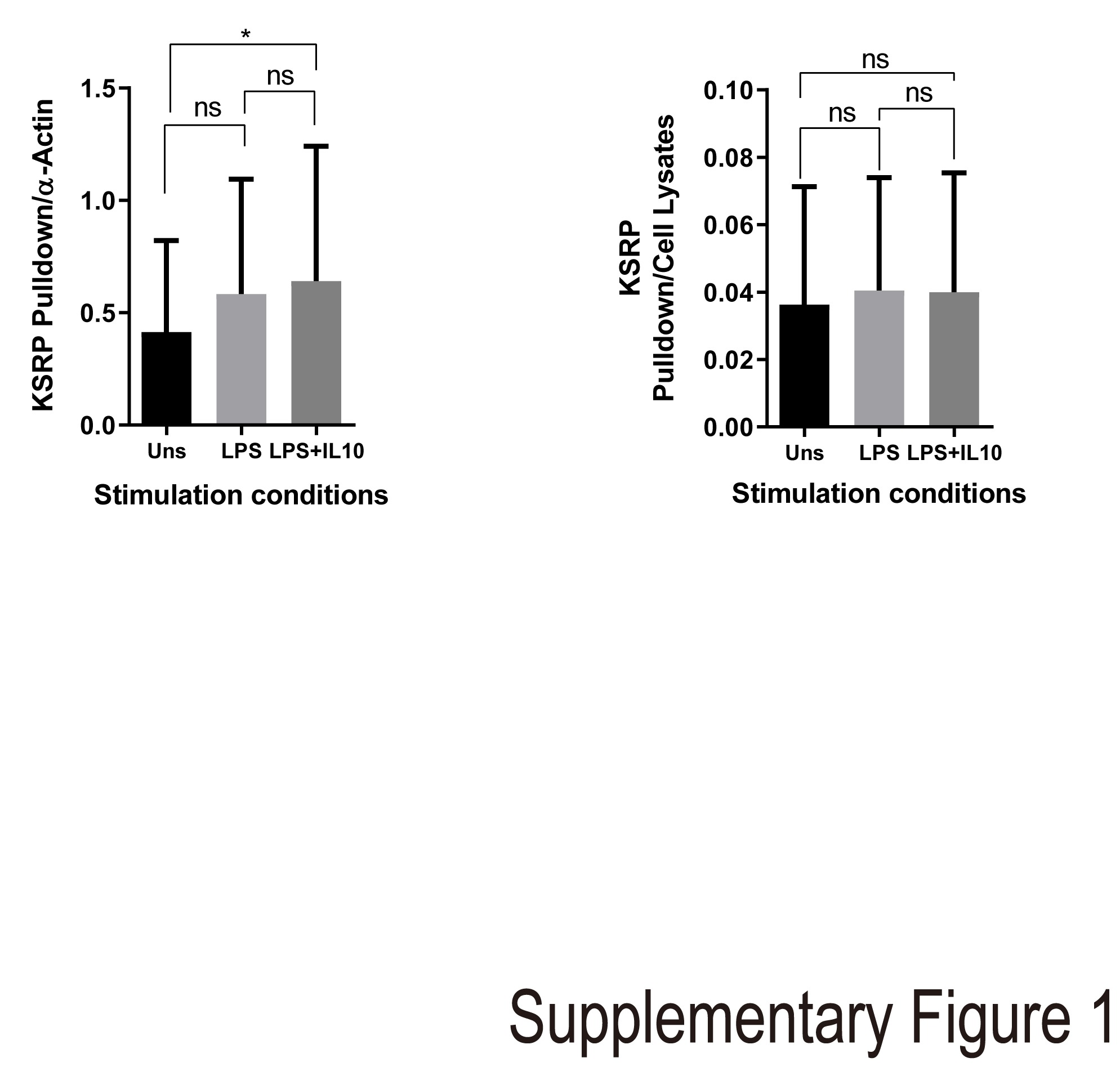

Supplement: S1 Fig — (TIF) [file pone.0231639.s001.tif]
