# Supplementary material for: Interleukin-10 control of pre-miR155 maturation involves CELF2
Source: PLoS One. 2020 Apr 23;15(4):e0231639. doi: 10.1371/journal.pone.0231639 (PMC7179890; doi:10.1371/journal.pone.0231639)
Supplement: S1 Raw images — (PDF) [file pone.0231639.s002.pdf]

“x” indicates lanes which were not included in the figure

Figure 1 A

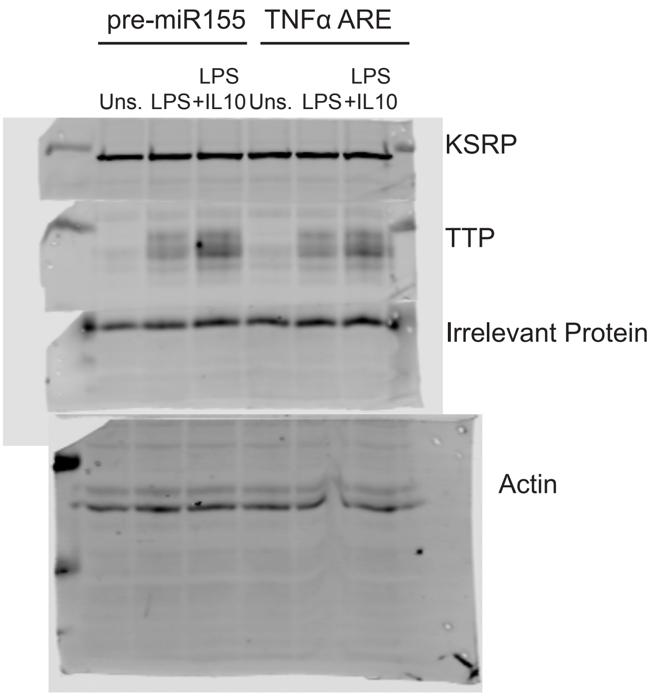

Figure 1 B

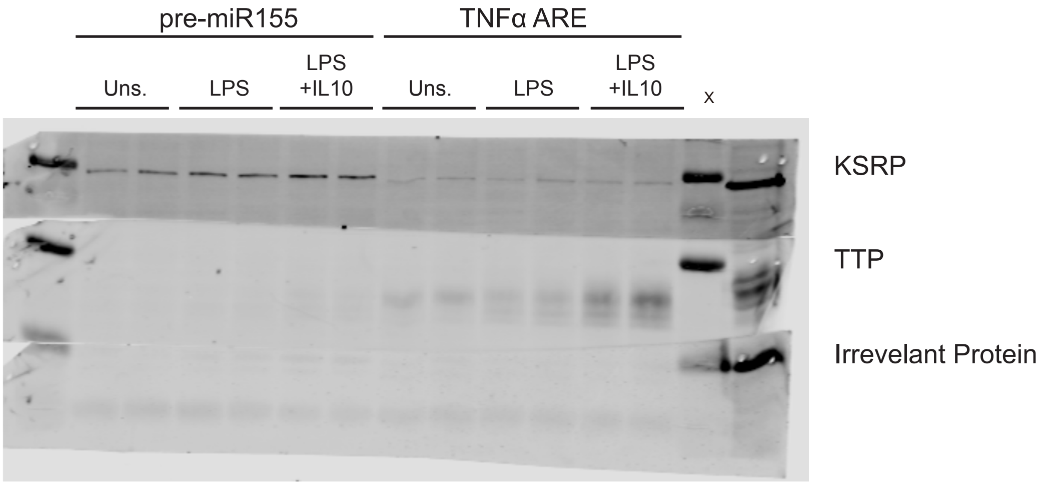

Figure 1

“x” indicates lanes which were not included in the figure

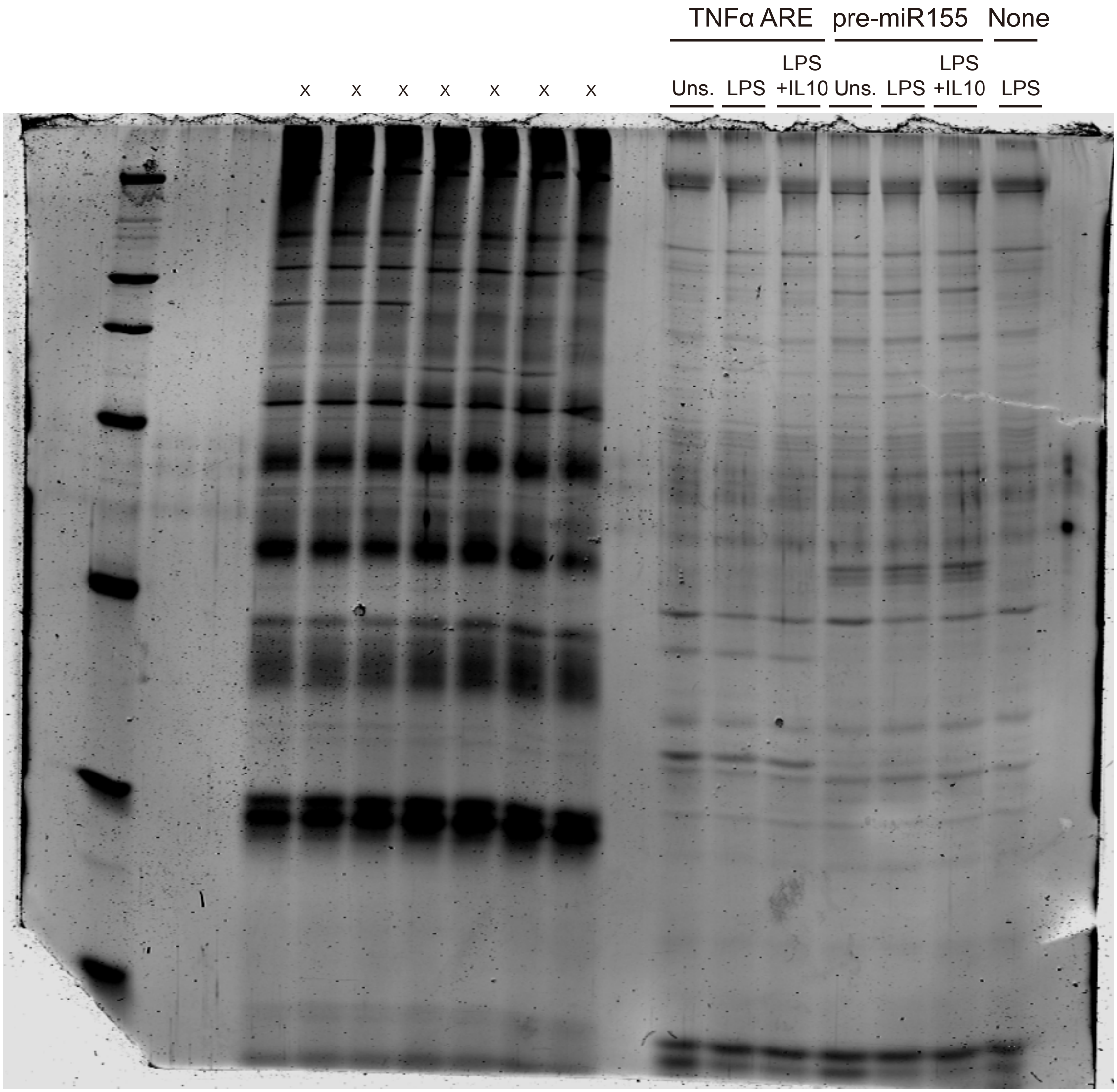

Figure 2

“x” indicates lanes which were not included in the figure

Figure 3A

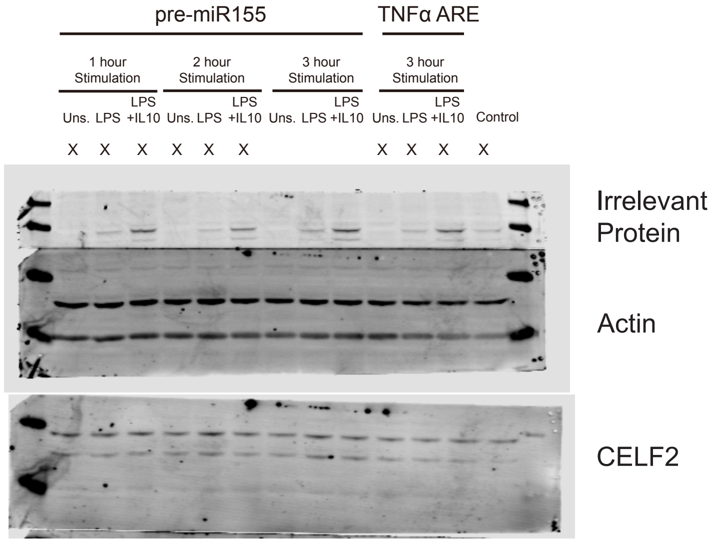

Figure 3B

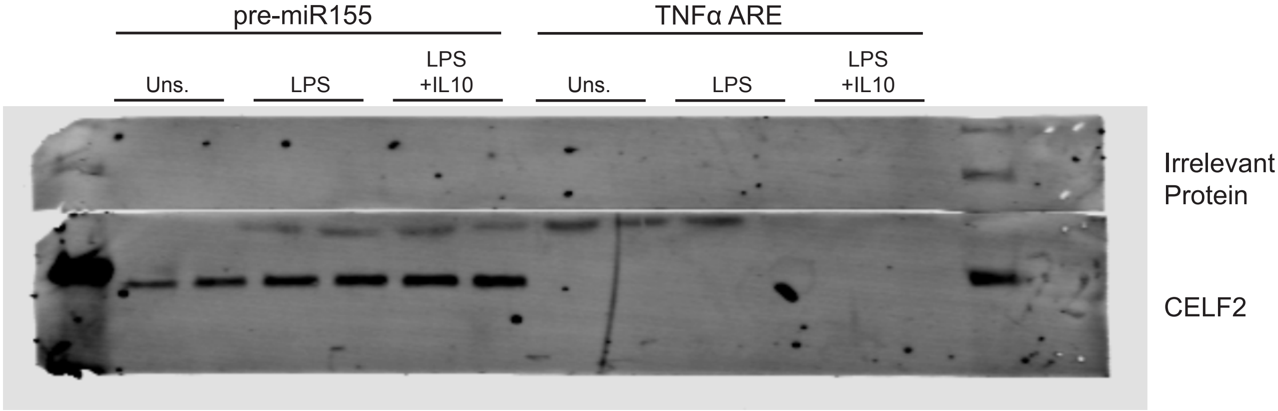

Figure 3

“x” indicates lanes which were not included in the figure

Two different CELF2 KD cell populations (1 and 2 generated from two different sgRNA targeting CELF2 gene) were generated. However, the knockdown of CELF2 protein was not stable in the CELF2 KD #2 cell population and these cells could not be used for further studies.

Exposure 1

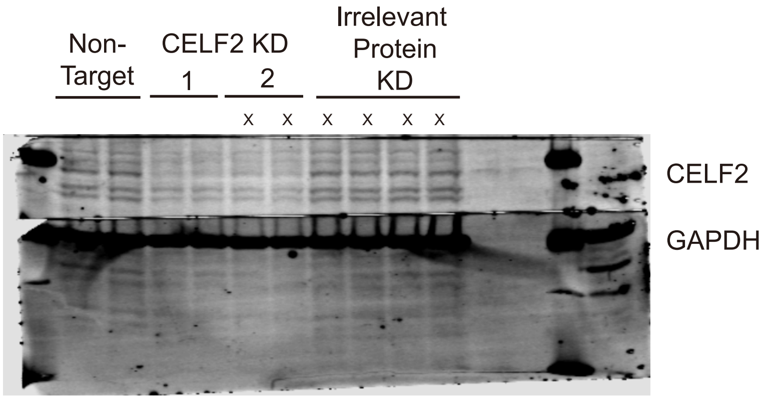

Exposure 2

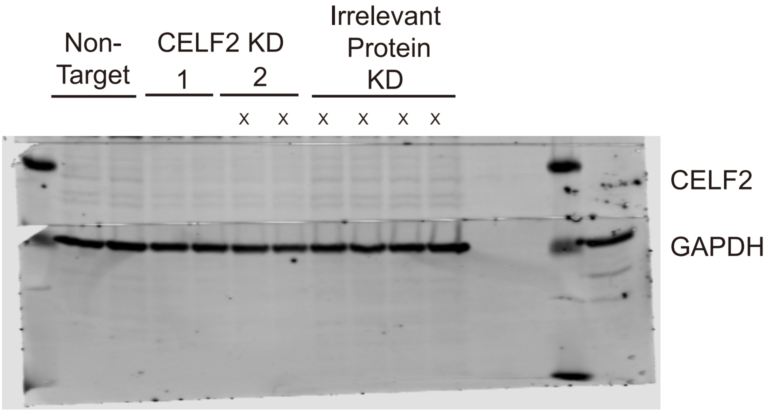

Figure 4
