# Supplementary material for: Interleukin-10 control of pre-miR155 maturation involves CELF2
Source: PLoS One. 2020 Apr 23;15(4):e0231639. doi: 10.1371/journal.pone.0231639 (PMC7179890; doi:10.1371/journal.pone.0231639)
Supplement: Attachment — Submitted filename: Response to Reviewers.docx [file pone.0231639.s003.docx]

As requested by the Editor, we have provided original uncropped and unadjusted images underlying all blot results reported in our figures. These images can be found within the file S1_raw_images.pdf within Supporting Information.

Reviewer #1: This concise study demonstrates that CELF2 binds to pre-miR155 and is important to the proper processing of pre-miR155 and regulation of TNFalpha. The data adds to the growing list of proteins that have been implicated in miR155 regulation and supports the authors conclusions. However, the authors should provide all of the mass spec data as there are many additional proteins that are also binding to pre-miR155 and this is important information for the community as well as being appropriate practice when a proteomics study is performed.

The mass spectrometry proteomics data has been deposited to ProteomeXchange via PRIDE database with the title “Identification of pre-miR155 binding proteins from macrophages treated with LPS or LPS + IL10”. The dataset is currently private, but can be accessed with the following reviewer account:

Username: [reviewer59427@ebu.ac.uk](mailto:reviewer59427@ebu.ac.uk)

Password: **IrTzQ6rb**

Identifier: PXD015750

The dataset will be publicly released later when the manuscript is published.

The information about the dataset and the database has been added to the “Acknowledgement” and “Method” section of the manuscript.

Reviewer #2: The authors Yoon et al. have very elegantly described the regulation of miR-155 biogenesis by LPS and IL-10 and have identified a novel interaction with CELF2 and pre-miR-155.
Minor comments,
Please provide high resolution images for all figures.
The authors should quantitate the western blots.
Line 408: TPP should be TTP.

Thank you for pointing out the misspelling of TTP protein. The misspelling has been corrected in the revised manuscript.

For the immunoblot images presented in the manuscript, all proteins were quantified and presented as graphs with the exception of the KSRP protein in pulldown (Fig. 1C). We now provide the quantification in graphs for KSRP in pulldown as Supplementary Figure 1 since KSRP is a control not central to the paper. If requested by the reviewer, we can include the KSRP quantification graph in main Figure 1. A summary of protein quantification graph locations is summarized in Table 1 below.

Table 1 – Description of immunoblot images and corresponding graphs

| **Immunoblot images** | **Protein** | **Location of quantification graphs** |
| --- | --- | --- |
| Figure 1A | TTP | Figure 1A |
|  | KSRP | Figure 1A |
| Figure 1B | TTP | Figure 1C, 1D |
|  | KSRP | Supplementary Figure 1 |
| Figure 3A | CELF2 | Figure 3A |
| Figure 3B | CELF2 | Figure 3C, 3D |
| Figure 4A | CELF2 | Figure 4A |

High-resolution images of the figure in TIF file format are available via links in the upper right corner of the figure images in manuscript PDF file.

Or use the following links below:

Figure 1:

<https://www.editorialmanager.com/pone/download.aspx?id=25477811&guid=349534b7-0af4-4470-96c6-aaaa94da1fa3&scheme=1>

Figure 2:

<https://www.editorialmanager.com/pone/download.aspx?id=25477812&guid=ac1b0815-6d04-4a52-81b1-e83ba7a8f311&scheme=1>

Figure 3:

<https://www.editorialmanager.com/pone/download.aspx?id=25477813&guid=2cf84fbd-6aa1-4a05-a565-e618f2d5336d&scheme=1>

Figure 4:

<https://www.editorialmanager.com/pone/download.aspx?id=25477814&guid=ed28a848-5847-408e-92b3-626d57b74ac2&scheme=1>

Figure 5:

<https://www.editorialmanager.com/pone/download.aspx?id=25477815&guid=fb43e883-52a5-4e83-955c-63174c11296c&scheme=1>
